# *Mycoplasma bovis* Infections—Occurrence, Diagnosis and Control

**DOI:** 10.3390/pathogens9080640

**Published:** 2020-08-06

**Authors:** Katarzyna Dudek, Robin A. J. Nicholas, Ewelina Szacawa, Dariusz Bednarek

**Affiliations:** 1 Department of Cattle and Sheep Diseases, National Veterinary Research Institute, 57 Partyzantów Avenue, 24100 Pulawy, Poland; ewelina.szacawa@piwet.pulawy.pl (E.S.); dbednarek@piwet.pulawy.pl (D.B.); 2 The Oaks, Nutshell Lane, Farnham, Surrey GU9 0HG, UK; robin.a.j.nicholas@gmail.com

**Keywords:** *Mycoplasma bovis*, cattle, disease, prevalence, control

## Abstract

*Mycoplasma bovis* is a cause of bronchopneumonia, mastitis and arthritis but may also affect other main organs in cattle such us the eye, ear or brain. Despite its non-zoonotic character, *M. bovis* infections are responsible for substantial economic health and welfare problems worldwide. *M. bovis* has spread worldwide, including to countries for a long time considered free of the pathogen. Control of *M. bovis* infections is hampered by a lack of effective vaccines and treatments due to increasing trends in antimicrobial resistance. This review summarizes the latest data on the epizootic situation of *M. bovis* infections and new sources/routes of transmission of the infection, and discusses the progress in diagnostics. The review includes various recommendations and suggestions which could be applied to infection control programs.

## 1. Introduction

In 2017, New Zealand became the last of the major cattle-rearing countries to be infected with *Mycoplasma bovis* [1]. Finland had also remained free until relatively recently but became infected via imported cattle in 2012 [2]. Undoubtedly, *M. bovis* is now the most important mycoplasma of livestock being a primary cause of mastitis, arthritis, keratoconjunctivitis and other disorders as well as a major player in the bovine respiratory disease complex (BRD) [3]. Previously *Mycoplasma mycoides* subsp. *mycoides,* the aetiological agent of the World Organisation for Animal Health (OIE)-listed contagious bovine pleuropneumonia, had this dubious distinction but this mycoplasma is now confined to countries in sub Saharan Africa.

**Figure 1:**
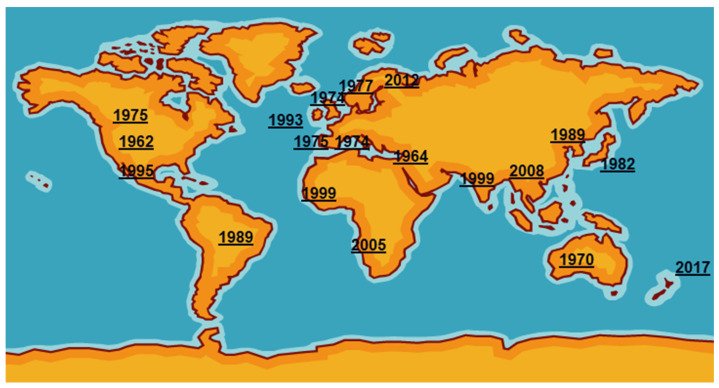
First detections of *Mycoplasma bovis* around the world.

*Mycoplasma bovis* was first reported in the USA in 1961 from a case of bovine mastitis then was probably exported in cattle of high genetic quality to Israel [3]. It then spread around the world, reaching the UK and the rest of Europe in the mid1970s (Figure 1). International trade in cattle and cattle products like semen has enabled its silent spread to all continents where cattle are kept. The date of isolation in a particular country, of course, is not necessarily the date of introduction even in the USA as mycoplasmas were very much an unknown quantity and their fastidious nature made isolation and detection an extremely difficult task. Indeed, it has only been in the last two decades with the introduction of DNA amplification techniques that detection and identification have become routine in many parts of the world. However, not all countries have veterinary diagnostic laboratories which can identify these organisms.

Initially the importance of *M. bovis*, particularly in BRD, was underestimated because of the promotion of more established and easier detectable organisms like the bacteria *Mannheimia haemolytica*, *Histophilus somni* and *Pasteurella multocida* and viruses, namely bovine respiratory syncytial disease, parainfluenza-3 virus, bovine herpesviruses, coronaviruses and bovine viral diarrhoea virus. The presence of *M. bovis* in healthy cattle, although at a much lower levels than infected ones, delayed recognition of its pathogenicity. Once the importance of environmental factors such as weather, variation in strain virulence and its interaction with the BRD pathogens were known, studies quickly demonstrated its widespread prevalence in pneumonic calves and, later, older cattle.

Despite attempts going back nearly half a century, control of *M. bovis* diseases is still problematic because of a lack of an effective commercial vaccine. Many have been marketed, particularly in the USA, but little data exist to assess their immunogenicity and protective properties [4]. To be valuable they are required to be part of multivalent vaccines incorporating the causative bacteria and viruses currently available for BRD. Presently, no vaccine is available for mycoplasma mastitis, a major problem in large dairy herds of North America where they are often untreatable. Indeed, the major trend in the last two decades has been the alarming decrease in susceptibility of *M. bovis* to the commonly used antimicrobials including the fluoroquinolones [5].

This review summarizes the latest data on the epizootic situation of *M. bovis* infections and new sources/routes of transmission of the infection and discusses the progress in diagnostics. The review also covers aspects related to *M. bovis* infection control, collecting various recommendations and suggestions which could be applied in the infection control programs.

## 2. *Mycoplasma bovis*: Key Facts

*Mycoplasma bovis* (*M. bovis*) is most often considered to cause caseonecrotic pneumonia, mastitis and arthritis [6,7]. However, cases of infectious keratoconjunctivitis, suppurative otitis media, meningitis, decubital abscesses, endocarditis and reproductive disorders have been associated with *M. bovis* [7,8,9,10]. Most importantly *M. bovis* is one of the causes of BRD with other aetiological agents, both bacterial and viral [11,12].

*M. bovis* is one of 13 species of mycoplasmas diagnosed in cattle; however, not all of them cause serious diseases, and some may even constitute normal flora of the bovine respiratory tract. For example, the most important mycoplasma in bovine severe respiratory diseases is the previously mentioned *Mycoplasma mycoides* subsp. *mycoides*. *Mycoplasma bovigenitalium* is generally associated with bovine reproductive disorders, while *Mycoplasma bovoculi* has been isolated from infectious keratoconjunctivitis in cattle [3]. *M. bovis* infections are non-zoonotic; however, substantial economic and cattle health and welfare impacts are felt worldwide [3]. *M. bovis* affects all age groups of cattle (prewean, postwean, neonate and adult) and all cattle sectors such as beef, milk or rearing [3]. *M. bovis* can persist in a herd for very long periods of time, with the possibility of pathogen shedding by the infected animals for a few weeks to several months [13,14]. The evolutionary absence of a cell wall in principle makes *M. bovis* resistant to penicillins and cephalosporins [3,4]. Moreover, in vitro studies on *M. bovis* field isolates show increasing trends in antimicrobial resistance, including tetracyclines and even newer generation macrolides considered effective against *M. bovis* infections [5,15,16,17,18]. *M. bovis* infections are usually characterized by chronic course and are difficult to treat successfully [3]. One recent in vivo study has shown an efficacy of treatment of the *M. bovis* pneumonia in calves using enrofloxacin given alone, unlike the combination therapy with co-administration of flunixin meglumine, a nonsteroidal anti-inflammatory drug or pegbovigrastim (immunostimulator), which rather exacerbated the disease. However, it should be remembered that fluoroquinolones, although effective in this case, should be used as antimicrobials of last resort [19]. Some experimental *M. bovis* vaccines have been shown to be immunogenic and protective; however, currently no commercial vaccines are available in Europe with only some autogenous vaccines in use in the United States and Great Britain [20,21,22].

## 3. Current Reports on the Epizootic Situation of *M. bovis*

It was previously reported that *M. bovis* has the ability to spread worldwide to countries for a long time considered free of the pathogen because of the widespread international trade in cattle [2,23,24]. The first case of *M. bovis* infection in Finland was recorded relatively recently in 2012 in pneumonic calves. In 2012–2015, 0.26% of Finnish dairy farms were *M. bovis* infected [2]. To date, it is estimated that only 0.8% of Finnish dairy herds were infected with *M. bovis* between 2012 and 2018 [23]. A two-year survey included 19 Finnish dairy farms previously free of *M. bovis* showed mastitis caused by *M. bovis* in over 89% of all farms tested; however, only a few clinical mastitis cases were seen. In the remaining two farms, no *M. bovis* mastitis cases were detected during the study period; calf pneumonia caused by *M. bovis* were, however, observed. In this study, the results may indicate a rather subclinical course of mastitis due to *M. bovis* infection. Additional data including *M. bovis* antibody detection using the MilA ELISA showed the majority of cows were positive for *M. bovis* throughout the study period, regardless of the infection status of the farm. It confirms that *M. bovis* may circulate for long time in the herd [23].

The detection of *M bovis* in New Zealand was remarkable for several reasons. First, New Zealand was probably the last major cattle-rearing nation to become infected; secondly, it does not import cattle, the main route of cross border infection, and had not done so for nearly a decade; and thirdly, New Zealand took the unprecedented decision to eradicate the organism from its cattle industry despite the fact the clinical disease was overwhelmingly mild. *M. bovis* was first detected in a dairy herd at the Bay of Plenty on the South Island in 2017. Since this isolation, up until June 2020, just over 1800 farms have been affected, involving the slaughter of nearly 160,000 cattle at a cost of NZ$203 million (about 116 million euros). With just over 250 farms still affected, complete eradication looks feasible but challenging and would be a first amongst cattle rearing countries. The origins of the outbreaks have still not been definitively traced but whole genome sequencing of 171 isolates from 30 infected herds indicated that the current outbreak was probably caused by recent entry of *M. bovis,* perhaps 1–2 years before detection, from a single source either as a single entry of a single *M. bovis* clone or, potentially, up to three entries of three very closely related *M. bovis* clones from the same source [25]; this suggests that there were probably several simultaneous outbreaks strongly implicating infected imported semen. Indeed *M. bovis* DNA was detected by PCR in one batch of semen but unfortunately could not be isolated. While analyses to date have not identified the source, the most closely related international isolates that have been characterised are European in origin [25].

Interesting information can be gathered by estimating on-farm/within-herd prevalence of *M. bovis* infections [26,27]. Such a repeated cross-sectional six-month study on *M. bovis* intramammary infections was conducted between 2017 and 2018 in four Estonian dairy herds with previously confirmed *M. bovis* positive status. The qPCR results of examination of pooled cow composite milk samples in the four endemically infected herds showed a differential and relatively low within-herd prevalence, which ranged between 0.4% and 12.3%. For the author, this could be a result of the different infection phases, *M. bovis* strain differentiation, intermittent shedding of the pathogen by the infected cows or low concentration of *M. bovis* in the examined milk samples. Similar prevalence (3.7–11%) was observed in clinical cases of mastitis due to *M. bovis* during a six-month study period in the four dairy herds. Additional evaluation of pooled cow colostrum samples during the same study period also showed low prevalence of *M. bovis* in the study herds ranging between 1.7% and 4.7% [26].

Within-herd prevalence of *M. bovis* DNA in cow colostrum samples was also estimated in 2016–2017 in seventeen Belgian herds with a recent infection of *M. bovis*. This survey was performed on dairy, beef and mixed-dairy farms with *M. bovis* positive status diagnosed less than one month before sample collection. The herds were additionally divided into two groups, depending on whether the infection was confirmed only in calves or in both calves and adult animals. The results showed only seven colostrum samples positive for *M. bovis* DNA originated from four herds, which was 1.9% of the total number of samples tested. In the positive farms on-farm/within-herd prevalence ranged between 2.8% and 30.0%, whereas the average within-herd prevalence estimated for all seventeen herds tested was 3.2%. According to the author, the reason for such low average within-herd prevalence of *M. bovis* DNA obtained in this survey was probably a result of differentiation in the infection phases in the periparturient cows or false positive results of real-time PCR assays used in *M. bovis* DNA detection particularly due to the possibility of ongoing co-infections with other *Mycoplasma* species [27]. In 2009, it was reported that 1.5% of all herd tested had bulk tank milk samples positive for *M. bovis* confirmed by culturing and PCR [28].

Data collected in Great Britain between 2006 and 2017 including diagnoses of respiratory disease, mastitis and arthritis due to *M. bovis* infections demonstrated a significant proportion of pneumonia (86.4%), which showed an increasing trend since 2014. The highest number of pneumonia incidents was diagnosed in 2017 (over 120 diagnoses), reaching 7.5% of all diagnosable submissions. For comparison, the annual cases of arthritis and mastitis for all the examined years were less than 30 per year, with a slight predominance for mycoplasma mastitis. In this survey the incidents of *M. bovis* pneumonia were diagnosed mainly in the postwean age group of calves. However, since 2012, the number of pneumonia diagnoses in the preweaning calves was comparable. The smallest number of *M. bovis* pneumonia cases was diagnosed in the neonate age group of calves. Seasonal data collected from 2006 to 2017 showed the largest number of respiratory diagnoses due to *M. bovis* were in the colder seasons, i.e., between October and March, which could be caused not only by temperature fluctuations, but also by closer contact of animals in the herd during housing [20,24]. Temperature fluctuations are probably related to stress accompanied by elevated blood corticosteroid concentrations, which may consequently predispose calves to *M. bovis* infection, as confirmed in both in vivo and in vitro studies using dexamethasone [29,30,31]. In the remaining months, i.e., from July to September, and from April to June, the respiratory submissions were comparable, although slightly higher in the spring months. Additional examinations also showed a higher incidence of *M. bovis* respiratory disease in the beef sector of cattle (almost 42%). Another slightly less affected cattle sector was dairy with 32.8% of *M. bovis* respiratory submissions [20]. A previous study performed in Great Britain between 1990 and 2000 showed that over 50% of a total of 1413 cattle isolates tested were *M. bovis*, mostly originating from pneumonia cases. *M. bovis* was also isolated from mastitis cases, joint fluid, eyes and sporadically from sheath washings, urogenital tract and heart blood [32].

The problem of subclinical intramammary infections with *M. bovis* as a consequence of recent clinical mastitis outbreaks in four Australian dairy herds was discussed in the study of Hazelton et al., which concluded that an early diagnosis of such cases may consequently prevent the future spread of *M. bovis* in the herd [13]. The apparent cow-level prevalence of *M. bovis* intramammary infections in these herds was determined immediately after cessation of outbreaks. Before the herd sampling between 2014 and 2016 all clinically affected cows due to *M. bovis* were culled. From a total of 2232 cows located in the main milking group of each herd from which 88 initial pooled milk samples were collected, only two *M. bovis* PCR positive cows were detected, which constituted less than 1% of average apparent cow-level prevalence of subclinical intramammary *M. bovis* infection. Additional tests performed individually on 15 cows located in the hospital group of each herd and *M. bovis* suspected gave five positive PCR results. *M. bovis* DNA was also detected by PCR in bulk tank milk collected from two study herds. However, in 6 out of 1813 cows from three study herds, *M. bovis* was isolated using microbiological culture. Five positive culture results were detected in cows located in the hospital group and *M. bovis* suspected, whereas the remaining one was from the main milking group, both within the same herd. For information, the culture positive cow in the main milking group had also positive *M. bovis* PCR result. In addition, *M. bovis* was isolated from bulk tank milk sampled from one study herd; however, it was not the same herd from which *M. bovis* culture positive cows were detected. To estimate *M. bovis* seroprevalence in the four study herds, a total of 199 sera were collected from 50 cows located in the main milking group of each herd, with the exception of one herd from which 49 results were estimated. The results showed the average *M. bovis* seroprevalence of 38%, which varied from 16% to 76%. It is also worth mentioning that in two of the four herds tested, several months after the herd sampling, new clinical cases or positive results in the hospital group bulk tank were reported, both confirmed by *M. bovis* PCR [13].

## 4. Disease Course and Source of *M. bovis* Infection

*M. bovis* infections occur with various clinical manifestations, such as pneumonia, mastitis, arthritis, otitis, keratoconjunctivitis, meningitis, endocarditis and others, the most important of which are summarized in Table 1. The clinical picture of respiratory disease diagnosed as *M. bovis* is not usually characteristic and often does not differ from clinical signs caused by infections with other bovine respiratory tract pathogens, especially in the presence of co-infections [20]. The study on feedlot beef calves showed that *M. bovis* was isolated from all diagnosed pneumonia categories, such as caseonecrotic bronchopneumonia, both caseonecrotic and fibrinosuppurative bronchopneumonia or fibrinosuppurative bronchopneumonia alone. In this study distinct synergism in pneumonia cases between *M. bovis* and *Pasteurellaceae* family pathogens, especially for *M. haemolytica,* was demonstrated. Both pathogens were identified in focal coagulative necrosis lesions within lung tissues [33].

In cases of keratoconjunctivitis as well as brain disorders, *M. bovis* infections, which are often overlooked in the differential diagnosis of these diseases, should be taken into account (Table 1).

As recently reported, both clinical and subclinical courses of mastitis due to *M. bovis* infection were detected [13,23]. However, the possibility of subclinical intramammary infections with *M. bovis* as a consequence of the recent clinical mastitis outbreaks should be considered as previously presented in the Section 3 in the study of Hazelton et al. [13].

It was first recognized that *M. bovis*-positive semen used in artificial insemination was a cause of mastitis outbreak in two naive dairy herds, despite high biosecurity and good farming practice carried out on these farms [2]. Out of the total of ten bulls used to inseminate cows with *M. bovis* mastitis diagnosed, only one of them appeared to be the *M. bovis* carrier. Additionally, only one of the cows from each herd that were inseminated with the contaminated processed semen from the same bull developed mastitis. In both study herds, the infection not only transmitted to other cows that were not inseminated with *M. bovis*-positive semen, but also to calves. The core-genome multilocus sequence typing (cgMLST) analysis of *M. bovis* strains isolated from the mastitis cases and the bull semen clustered together [2].

The role of airborne transmission of *M. bovis* is unclear with little experimental evidence supporting this route of infection [37,38]. In response to exposure of calves to aerosolized *M. bovis*, respiratory disease was induced. In the infected calves, specific *M. bovis* lung lesions confirmed by necropsy and histological examinations were observed despite the lack of clinical signs. However, re-isolation of *M. bovis* from the upper trachea in most infected calves was additional confirmation of this infection route [37].

Recent reports on *M. bovis* indicated colostrum as a possible source of infection based on positive results for *M. bovis* DNA [26,27]. Additionally, in one of these studies, herd-specific *M. bovis* strains were isolated from cows with clinical mastitis and calves affected with respiratory disease showing possible transmission of the pathogen between dairy cows and calves via contaminated milk. However, in this study other routes of *M. bovis* infection transmission like direct/indirect contact between animals within the study herds, animal handling or air-borne route cannot be excluded [26]. The most important sources of *M. bovis* infection/routes of *M. bovis* infection transmission are summarized in Table 2. Other no less important sources/routes of *M. bovis* infection transmission not included in the Table 2 such as nose-to-nose contact between animals or fomites (e.g., farm-personnel’s contaminated hands, equipment), although difficult to directly prove or document, should also be considered [26,39,40].

Within the host, *M. bovis* disseminates by the haematogenous route, which may result in subsequent lesions in organs other than those initially affected. In one such study all diagnosed cases of arthritis in feedlot beef calves were accompanied by lung lesions, which accounted for nearly 50% of all diagnosed *M. bovis*-related pneumonias. The arthritis cases were probably of pulmonary origin [33]. In post-mortem findings in *M. bovis* affected calves, both meningitis and otitis media/interna were diagnosed. In other calf necropsy examinations, necrosis within the brain and fibrinous heart lesions due to *M. bovis* infection were evident [36]. The ability of *M. bovis* to spread within different organs of the same host was previously confirmed [7]. In the majority of calves diagnosed with suppurative otitis media severe lung lesions were observed. In some of them cerebellar meningitis was also diagnosed. Additionally, in some calves, *M. bovis* antigen was identified in the temporal bone, liver and kidney [7].

## 5. Currently Used Diagnostic Methods

The clinical signs of infections in cattle associated with *M. bovis* are non-specific; for that reason, sensitive, accurate and rapid testing of animals is needed for reliable diagnosis. Culturing of *M. bovis* is a gold standard method but is time-consuming and requires specific conditions. Different kinds of media are widely used in experimental studies and in confirmation of infection caused by *M. bovis*, and include Hayflick’s [42], modified PPLO [43] and Eaton’s [44]. Mycoplasmas are fastidious, slow growing and can be easily overgrown by other bacteria. During the last few years various tests have been used for the detection of *M. bovis* infections in cattle (Table 3).

### 5.1. Real-Time PCR Assays for M. bovis Detection

Detection of *M. bovis* by real-time PCR preceded by culture enrichment of the samples improves detection when DNA is present at low concentrations. Furthermore, a selective broth-enrichment step increases the probability of *Mycoplasma* recovery when compared to direct plating on agar [55]. In the real-time PCR assay [45], milk samples from dairies and lung tissue samples were culture-enriched in PPLO broth for 24 h before analysis. In another qPCR for *M. bovis* testing [46], the nasopharyngeal swabs were cultured for 3–5 days before the analysis. The molecular methods are optimized for the detection of *M. bovis* in nasopharyngeal swabs and milk samples, but they can be optimized to be used for the detection of *M. bovis* in different specimens [2,26,27,48,49]. In 2020, a qPCR was developed for the detection of *M. bovis* in tracheal aspirate samples derived from calves [49]. In research on *M. bovis* intramammary infection, the presence of this pathogen in colostrum and additionally in milk from clinical cases was assessed with qPCR [26]. It is also possible to detect *M. bovis* in processed semen [2,48]. The real-time PCR assays are characterised often by a low limit of detection (LOD) and specificity near to 100% [45,46,47,48]. Taking into consideration that the number of mycoplasmas that are shed during the infection is about >1 × 10^6^ CFU/mL in milk [4] and the LOD for real-time PCR for *M. bovis* detection in milk is 1.3 × 10^2^ CFU/mL [48], the probability of the detection of infected cow in a herd is high. To assess the best sensitivity, the real-time PCR assays for *M. bovis* detection are usually used after an enrichment procedure of the samples. Additionally, centrifugation of the milk and plating the resuspended pellet of bacteria improves detection of mycoplasmas with culture. After such treatment, it was four times more likely to detect of a positive sample when compared to traditional culture regarding very small concentrations [56]. The combination of culture of viable bacteria and qPCR results enables the most accurate confirmation of active infection in animals.

### 5.2. Fast and Cost-Effective Assays for M. bovis Detection

Another approach for *M. bovis* detection is to design a simple and cost-effective assay run at a single temperature without the need of using specific equipment, which will be useful to process in developing countries. LAMP is recently of interest because it enables results to be received quickly, and the reaction is normally completed in less than 2 h; furthermore, there is no need to have expensive laboratory equipment, as it is performed at a single temperature [57]. LAMP gives better results than qPCR when performed on purified DNA but is susceptible to contamination. Two assays, namely LAMP and qPCR developed for *M. bovis* detection in milk samples from individual cow quarters and bulk tank milk samples, accurately detected *M. bovis* isolates but gave false positive results for one *Mycoplasma bovigenitalium* isolate [47]. Another method called isothermal DNA amplification assay, a technique based on recombinase polymerase amplification (RPA) with lateral flow dipstick (LFD), allows one to obtain the result in 30 min and is dedicated for *M. bovis* DNA extracted directly from clinical samples i.e., nasal swabs, lungs tissue samples, joint fluids and bulk tank milk samples; no cross-reactions were observed with other *Mycoplasma* species [53]. Usually, LAMP assays are more sensitive than end-point PCRs, for example high sensitivity and specificity for all milk sample types was obtained with the use of LAMP combined with a procedure for ultra-rapid extraction (PURE-LAMP), in which various sample types i.e., bulk tank milk, mature milk, colostrum/transitional milk and mastitis milk were examined [52]. Similar parameters were obtained in LAMP for the examination of *M. bovis* in milk from mastitis cases [51].

### 5.3. Immunohistochemistry and In-Situ Hybridization

Although molecular methods are advantageous, they can only provide the data on *M. bovis* DNA, and there is lacking information about the presence of viable bacteria. Immunohistochemistry (IHC) and in-situ hybridization (ISH) are types of techniques which have the advantage that they are able to detect the localization of *M. bovis* antigen or DNA, respectively, in the examined tissue of the infected animals [12,19,41,58,59]. The IHC used in the study on calves experimentally infected with *M. bovis* allows one to detect *M. bovis* antigen in the bronchiolar epithelial cells in the lung tissue with histopathological changes that are characteristic for bronchiolitis [19]. Results of another experiment proved that *M. bovis* antigen was detected on the surface and inside the cytoplasm of bronchiolar epithelial cells in the pneumonic foci and in the cytoplasm of phagocytes at the margin of bronchiolar exudates [58]. In the study on aborted foetus and neonatal calf that were infected with *M. bovis*, its antigen was found with the use of IHC in the brain, liver, lungs and placenta of aborted foetus, and ISH showed the presence of its DNA i.e., in lungs and placenta of the examined animals [41]. The research on long-term survival of *M. bovis* in tissues of infected calves showed the persistence of this pathogen in necrotic lung lesions several weeks after the infection with the use of both methods [59]. It is also possible to examine the pulmonary samples of calves with BRD. IHC was used to detect the *M. bovis* antigen intralesional in different areas of the lungs [12]. However, while these techniques allow one to obtain significant information, they are also expensive and labour intensive and require trained staff.

### 5.4. A Matrix-Assisted Laser Desorption/Ionization Time-of-Flight Mass Spectrometry for M. bovis Detection

The matrix-assisted laser desorption/ionization time-of-flight mass spectrometry (MALDI-TOF MS) procedure has been applied to *M. bovis* detection. It was optimised for the detection of *M. bovis* isolates and found to be a suitable test for routine diagnostics in cattle, especially those from BRD cases. The protocol enables the identification of *M. bovis* from bronchoalveolar lavage fluid (BALF) after enrichment in culture. The higher number of positive samples was obtained after 72 h of enrichment. The main advantage of MALDI-TOF MS is that it only detects viable bacteria, which indicates that cattle have active rather than historic infections [54].

### 5.5. Molecular Typing

The analysis of *M. bovis* isolates with typing and sequencing methods can give additional information about their relationships and evolution. The multilocus sequence typing (MLST) analysis was proved to be suitable for molecular typing of *M. bovis* and the assessment of geographical relatedness of isolates. The MLST scheme based on eleven housekeeping genes was evaluated. Three genes, *dnaN*, *metS* and *hsp70*, were taken for the sequence analysis and the remaining eight genes, i.e., *adk*, *efp*, *gmk*, *gyrB*, *polC*, *rpoB*, *tpiA* and *uvrC* were not chosen for the further analysis. It allows the acquiring of information on sequence variation, its type of distribution and disappearance of some sequence types [60]. A later study [61] assessed two MLST schemes for *M. bovis* isolate typing. The comparison of the performance of the two MLST schemes and additional identification of a new reference scheme capable of full typing of the examined isolates was made. The PubMLST reference method contains *adh-1*, *gltX*, *gspA*, *gyrA*, *gyrB*, *pta-2*, *tdk* and *tkt* locus; it is thought to be discriminatory and informative enough, but in this study, *adh-1*, one of the typing loci of *M. bovis* isolates, was missed. According to this reference scheme, the *adh-1* locus should be retired from the analysis. This approach was not beneficial for the study because the discrimination index received with the use of the six remaining PubMLST loci failed to reach the benchmark recommended for a reference method, and the addition of a seventh locus had to be made. The alternative scheme contains seven loci: *aptA*, *dnaA*, *metS*, *recA*, *rpoD*, *tkt* and *tufA*. The comparisons of examined *M. bovis* genome sequences identified the *dnaA* locus from the alternative scheme as the optimal replacement for *adh-1*.

Another approach for epidemiological studies is the use of whole genome sequencing (WGS) to evaluate the molecular epidemiology and genomic diversity of *M. bovis* isolates as well as their genetic relationship. The single nucleotide polymorphism (SNP) analysis can be used to assess the intraspecies relationship and the presence of a dominant genotype that can be associated with one type of disease. This study is relevant to better understand the global epidemiology of this important pathogen and to assess control strategies [62]. Comparison of the *M. bovis* sequences can be used in assessing the genetic diversity of the strains [63] or to get the information about gene virulence [64].

WGS was used in New Zealand to track the outbreaks first identified in 2017. In all, 171 isolates from 30 infected herds have so far been sequenced, and results indicate that the current outbreak was probably caused by recent entry of the mycoplasma, perhaps 1–2 years before detection, from a single source either as a single border crossing of a single clone or, potentially, up to three border crossings of three very closely related clones from the same source (TAG 2019) probably in germplasm imported from Europe.

### 5.6. Serological Approaches

Serological diagnosis based on detection of specific antibodies to *M. bovis* is suitable and practical for the assessment of prevalence and epidemiological studies of herds [39]. Although serological testing is a reliable method for identification of infected animals, specific antibodies do not appear until 10 to 14 days after the infection but remain elevated for several months [65]. Various indirect ELISAs are used for anti-*M. bovis* antibody detection in cattle herds. The BIO K302 ELISA (BioX Diagnostics) was applied for evaluation of antibody response to *M. bovis* in serum and milk samples [13,66,67]. A study conducted in Belgium [67] showed that the ELISA is able to detect *M. bovis* specific antibodies in bulk tank milk up to 12 months after the outbreak of the disease. Researchers [66] examined bulk milk tank samples for all Danish herds with this ELISA and concluded that the cut-off value should be increased from 37%, as suggested for animal-level diagnosis, to 50%, to obtain more adequate sensitivity and specificity for bulk tank milk analysis. On the other hand, as a result of a European inter-laboratory comparison conducted on 180 serum samples, the sensitivity and specificity of BIO K302 ELISA was determined to be 49.1% and 89.6%, respectively [68]. However, in 2020 it was confirmed that this ELISA was suitable for the serological evaluation of anti-*M. bovis* antibodies in longitudinal studies. Despite the low number of apparent clinical mastitis cases, it was useful in evaluation of *M. bovis* seroprevalence in dairy herds, which was on average 38% (16–76%), as mentioned before [13].

Another indirect ELISA, made in-house and based on a fragment of a recombinant mycoplasma immunogenic lipase A (MilA), was developed [69]. This assay can be also useful for bulk tank milk sample analysis. The results of the presence of anti-*M. bovis* antibodies in bulk tank milk were positively correlated with the antibody detection in sera of the examined animals. Additionally, there was made a comparison between BIO K 260 (BioX Diagnostics) and the MilA ELISA [23], and the latter test gave a higher number of positive samples for *M. bovis*, and they were more convergent with those obtained with culture or real-time PCR. The obtained sensitivity and specificity for this test was 94.3% and 94.4%, respectively. Additionally, it was shown that the MilA ELISA is also suitable for testing the presence of anti-*M. bovis* antibodies in the early stages of calf life (from the 3rd week of life) [70].

### 5.7. Interlaboratory Trials of Diagnostic Tests

*M. bovis* causes serious health problems in cattle herds almost all over the world, but its detection is not harmonised as yet and relies on different diagnostic methods, often in-house molecular techniques based on a variety of target genes and various different DNA extraction methods. There was conducted a European interlaboratory comparison of the diagnostic utility of the molecular tests for *M. bovis* detection [71]. Six laboratories from different countries were included in the study. Five different DNA extraction methods from bacterial culture and BALF samples were used. The molecular tests were made with the use of seven different PCR assays based on *polC*, *oppD*, *uvrC* and V4-V4 16S rRNA target genes. The comparison revealed that although the research used various assays, they had comparable diagnostic utility for *M. bovis* detection in cattle. The analytical specificity of the different PCR methods was comparable for all of the laboratories, except one, where *M. agalactiae* was detected because of the use of 16S rRNA target gene. The LOD was from 10 to 10^3^ for the real-time, and from 10^3^ to 10^6^ CFU/mL for the end-point assays. According to the authors, this difference was acceptable. Cultures correctly detected the presence of *M. bovis* in bronchoalveolar lavage fluid samples and were consistent with PCR results. The recent comparison of diagnostic methods used in the different veterinary laboratories fortunately showed consensus.

### 5.8. Mixed Infections

Other *Mycoplasma* spp. can also be associated with *M. bovis* infections in cattle. In BRD cases, most often *M. dispar*, *M. canis* and *M. arginini* are implicated [3,72]. In mastitis mycoplasmatica and reproductive disorders, *M. bovigenitalium*, *M. californicum* and *M. alkalescens* can also participate [73,74]. A test based on PCR with the 16SrRNA target gene and separation of the PCR products using denaturing gradient gel electrophoresis (PCR–DGGE) enabled the differentiation of 13 *Mycoplasma* spp. of bovine origin in mixed infections [75]. Traditionally, culture is used for the confirmation of BRD infections, but the incubation period for each examined bacterial pathogens is different and samples inoculated onto agar plates are often overgrown with other, fast growing bacteria. For that reason, the multiplex real-time PCRs used by the laboratories [49,50,76] are the most suitable for simultaneous direct detection of *M. bovis* and other pathogens involved in BRD, such as *P. multocida*, *M. haemolytica* and *H. somni*, in contrast to methods not dedicated for different pathogen identification in mixed infections such as one-target PCR, traditional culture or MALDI-TOF MS [77]. When using one target PCR, there is no information about the involvement of other pathogens in the disease, different bacteria have various growth requirements and slow growing bacteria can be easily overgrown by others, and MALDI-TOF MS is not able properly detect all organisms from polymicrobial samples.

Various diagnostics methods for fast and accurate detection of *M. bovis* in various sample types and typing methods for identification and analysis of its strains in the last few years have been developed for evaluation of the disease course. Methods should be chosen according to the purpose of the survey, for herd-level testing or for individuals, or should be considered in terms of its usage for the specimen. The use of a combination of molecular, serological and culture-based methods is necessary for reliable diagnosis of diseases caused by this pathogen in cattle.

## 6. Control—Recommendations for *M. bovis* Control Programs

Due to the lack of efficient vaccines against *M. bovis* and increasing trends in antimicrobial resistance of *M. bovis* field isolates, it is important to provide consistent, possibly unified rules for effective control and/or eradication of *M. bovis* infections. However, in many ways, preventing the spread of *M bovis* into healthy herds is relatively easy, as the screening of small numbers of cattle from source herds by serological tests, such as ELISA, can ensure that herds remain free of disease; this was successfully achieved in the Republic of Ireland when the national herd free of *M. bovis* was restocked following the BSE crisis [78]. Whether the Irish national herd is still free is unknown. However, few countries have active eradication plans for *M. bovis*, and because of its presence in all cattle-rearing countries, it is not subject to OIE regulations; indeed, it is very difficult for countries to impose trade restrictions when they themselves are infected. Israel has attempted to identify countries that export infected livestock into their country by mass screening between 2010–2011 and found cattle from Lithuania, Hungary and Australia to be highly seropositive [79].

Undoubtedly the most ambitious and unique plan for the complete eradication of *M. bovis* was made in New Zealand where infection was first recognised in 2017. The decision was made to cull infected and contact cattle when the number of infected farms was low but now remains increasingly challenging though still feasible according to Technical Advisory Group in 2019 [25] because of the high number of infected farms traced subsequently. To date over 2000 infected farms have been traced, although most without clinical or gross pathological signs. Detecting infected farms proved difficult at first because of the use of relatively insensitive diagnostic tests, but now serological ELISA testing bulk tank milk is being used in parallel with real-time PCRs. This has increased confidence that eradication can be achieved, although the process is likely to take at least 5 years or maybe longer.

In Finland, there is a voluntary *M. bovis* control program (Animal Health ETT) for cattle farms since 2013, which four years later associated 75% of all dairy farms [2,23].

Pasteurisation or heat treatment is one of proposals to eliminate the risk of *M. bovis* shedding via colostrum or raw milk. Another alternative may be to avoid pooling of colostrum within endemically infected farms, discarding colostrum originating from *M. bovis* affected cows, or colostrum purchasing as replacer [27]. As previously documented, a commercial on-farm pasteurizer was able to destroy *Mycoplasma* spp. tested in 71.7 °C for 15 s, including *M. bovis*. Additional data showed an average 25% reduction in total immunoglobulin concentration in colostrum after 30 min pasteurization, from 22% at the low temperature range (63.9–66.7 °C) to 27% at high temperatures (68.3–70.8 °C) [80]. However, heat treatment of colostrum may affect cytokine absorption and immune response in neonatal calves. A reduction in the circulating IL-1β in dairy calves fed colostrum heat-treated to 60 °C for 60 min was demonstrated, although without affecting other immune parameters tested such as IFN-γ or IgG concentrations [81].

The generally recommended rule to control subclinical intramammary infections due to *M. bovis* is sampling of cows with high somatic cell counts (SCC) in milk; however, as was shown in some studies, cows with no clinical signs of mastitis and low SCCs (<200,000 cells/mL) can be *M. bovis* positive [13,82]. However, these differences may be a result of the disease stage. The study of Kauf et al. [83] showed that infusion of a mastitic *M. bovis* strain in one quarter of ten first-lactation cows with milk SCCs of <200,000 cells/mL caused initial increase in mean milk SCCs within 66 h post infusion. During the study period, the SCC counts fluctuated, with a peak value of 119.82 × 10^6^ cells/mL at 90 h following the infusion; however, they persisted at a higher level than the control until the end of the study at 240 h post infection [83].

It was recommended that clinically affected *M. bovis* cows should be separated and moved from the main milking group to hospital or another group to prevent the infection spread in the herd. According to the author’s opinion, cows within main milking group should be constantly monitored via bulk tank milk testing [13]. However, there was evidence of *M. bovis* mastitis incidence and transmission in the hospital pen following the introduction of cows with *M. bovis* clinical mastitis from three different milking pens, which should not be underestimated [84]. Bulk tank milk testing seems to be effective due to previously reported mycoplasma shedding via milk of cows with mastitis at above 1 × 10^6^ CFU/mL [4]. It was suggested that if a positive result is obtained in bulk tank milk testing, it is a good strategy to follow up with pooled milk samples from five cows to identify the individuals [85]. However, SCC screening in bulk tank milk for *M. bovis* infection control does not appear to be effective [13]. An important suggestion for programs designed for *M. bovis* mastitis control is milk testing of newly introduced animals into the lactating herd. Additionally, using antibiotics to treat *M. bovis* mastitis should be discouraged [4].

One recommendation for *M. bovis* control programs is to combine regular monitoring of mastitic cows and pneumonia calves with bulk tank milk testing and longitudinal screening of young stock in herds [23].

Another option in the prevention/eradication of *M. bovis* infections is farm sanitization using effective disinfectants. Only a few studies on disinfectant efficacy in inactivating *M. bovis* has been undertaken. The most recent study estimated the efficacy of different dilutions of citric acid and sodium hypochlorite against *M. bovis*. The results showed that the acceptance criterion for an effective disinfectant of 10^6^ fold reduction in the *M. bovis* viability was met for 0.5% citric acid and 1% sodium hypochlorite in the presence of organic material. However, in the absence of organic material, a 10^6^ fold reduction in the *M. bovis* viability was observed for 0.25% citric acid and 0.04% sodium hypochlorite [86]. In another study, the efficacy of five different classes of teat dips were tested against *M. bovis* in the context of their use in maintaining pre- and post-milking hygiene and preventing *M. bovis* mastitis. All of them showed germicidal activity against *M. bovis*, but the iodine-based formulation was the most effective in this study [87].

To reduce the risk of *M. bovis* shedding in semen, it is worth paying more attention to the type and volume of antibiotics added to seminal extenders, because currently used mixtures have a more bacteriostatic rather than bactericidal effect on *M. bovis*. According to the author, the antibiotic combination in seminal extenders should be re-evaluated or alternatively *M. bovis* testing in processed semen should be performed [2].

Above all, it is important to recognize the subclinically infected cattle, which can be facilitated by regular monitoring/screening of different age groups of animals using various methods to prevent uncontrolled *M. bovis* shedding [23].

In summary, *M. bovis* infections are difficult to control/eradicate most of all due to the intracellular nature of the pathogen and biofilm production, which effectively hamper disease treatment. Additionally, increasing trends in antimicrobial resistance of field *M. bovis* isolates reduce the effectiveness of the therapy used routinely for *M. bovis* infections. The high genetic and antigenic variability of field *M. bovis* strains makes them easier to avoid the host immune response. In addition, the general chronic nature of the disease facilitates the spread of the mycoplasma in the herd. Additionally, the lack of effective vaccines makes the eradication of *M. bovis* infections very difficult from cattle population. The relentless and silent spread of *M. bovis* into the infection-free areas is also a feature of this disease. Therefore, regular monitoring/screening of different age groups of animals should be applied, especially for early detection of subclinical carriers in cattle herds; work is also required to develop effective vaccines to provide suitable control of *M. bovis* infections. Finally, there is also an urgent need to develop uniform recommendations that will be included in the programs designed for *M. bovis* infection control.

## Figures and Tables

**Table 1:** Examples of clinical manifestations of *M. bovis* infections. The sequence presented is consistent with the frequency of each clinical manifestation from the most to the least frequently diagnosed cases in cattle.

Course of *M. bovis* Infection	Type of Research (Experimental/Survey)	Cattle Sector	Main Clinical Signs/Lesions/Subclinical	Methods Used for the Infection Confirmation/Presence	Reference
pneumonia	survey	beef	caseonecrotic bronchopneumonia; fibrinosuppurative bronchopneumonia	IHC; PCR	[33]
	experimental	dairy-cross	nasal discharge; coughing; caseonecrotic pneumonia	ELISA for *M. bovis* antigen detection; IHC; ELISA for specific antibody detection	[19]
mastitis	survey	dairy	clinical mastitis; subclinical mastitis	culture; real-time PCR; two different ELISAs for specific antibody detection (MilA IgG ELISA; BioX ELISA)	[23]
	survey		clinical mastitis; subclinical mastitis	culture; PCR; ELISA for specific antibody detection	[13]
arthritis	survey	beef	arthritis; tenosynovitis	culture; passive hemagglutination test	[33]
	experimental	dairy	joint swelling; lameness/fibrinosuppurative synovitis and tenosynovitis; thrombus presence	culture; indirect hemagglutination test	[34]
otitis	survey	dairy	ear droop; otic exudate	ELISA for specific antibody detection; DGGE	[35]
	survey	beef	ear droop; exudative otitis media; facial paralysis; occasionally nasal exudate; nystagmus, head tilt, ataxia/suppurative lesions in the middle ear; lung consolidation (most cases); cerebellar meningitis (some cases)	culturing; immune-peroxidase test; PCR; IHC; transmission electron microscopy	[7]
kerato-conjunctivitis	survey	beef	“pink eye” signs	culture; RAPD; PCR-RFLP; DNA sequencing	[8]
brain disorders	survey	dairy	head tilt; central nervous system signs/purulent meningitis	ELISA for specific antibody detection	[36]
			lethargy, blindness; teeth grinding/cerebral hemisphere necrosis	enrichment and capture ELISA	
endocarditis	survey	beef	no clinical signs; caseated lesions in the heart	culture; *uvrC* gene PCR; loop-mediated isothermal amplification assay; IHC	[10]

**Table 2:** Examples of sources of *M. bovis* infection/routes of *M. bovis* infection transmission.

Source of Infection/Route of Infection Transmission	Type of Research (Experimental/Survey)	Cattle Sector	Number of Herd/Farms Tested	Methods Used for the Infection Confirmation/Detection	Reference
colostrum	survey	dairy	4	qPCR	[26]
	survey	dairy, beef and dairy-mixed	17	real-time PCR	[27]
milk	survey	dairy	4	qPCR; culturing; core-genome multilocus sequence typing (cgMLST)	[26]
semen	survey	dairy	2	culturing; real-time PCR; WGS; cgMLST analysis	[2]
air-borne	experimental	dairy-cross	not applicable	culturing; *polC* PCR; MilA IgG ELISA; *post mortem* examination; histopathological examination	[37]
intrauterine	survey	dairy	not described	culturing; IHC; ISH	[41]

**Table 3:** The characteristics of recently developed methods for *M. bovis* detection in various specimens from cattle.

Assay/Target	Samples	Limit of Detection	Sensitivity	Specificity	Reference
real-time PCR*/uvr*C	lung samples (n = 30); milk samples (n = 21)	100 fg DNA; 40 genome copies/reaction; 250 CFU/mL	10^3^-fold more sensitive than conventional PCR	100% (evaluated for 6 *Mycoplasma* spp. and 6 species of bacteria)	[45]
qPCR*/uvr*C	deep nasopharyngeal swabs (n = 208)	1.61 × 10^2^ CFU/mL	100%	87.27%	[46]
qPCR*/glt*X	milk samples from individual quarters (n = 9); bulk tank milk samples (n = 59)	10–100 genome equivalents/reaction; 1 × 10^4^–1 × 10^5^ cells/mL	100%	94.4% (evaluated for 3 *Mycoplasma* spp.)	[47]
real-time multiplex PCR *M. bovis/uvr*C *M. californicum*/*rpo*B *M. bovigenitalium*/16S–23S rRNA	swab samples (n = 95); semen samples (n = 44); individual milk samples (n = 114); bulk tank milk samples (n = 221)	1.3 × 10^2^ –1.3 × 10^7^ CFU/mL	not applicable	100% (evaluated for 10 *Mycoplasma* spp. and 11 species of bacteria)	[48]
multiplex qPCR Pneumo 4B/*M. bovis* *M. haemolytica* *P. multocida* *H. somni*	tracheal aspirate samples (n = 176)	10 genome copies; 1.1–3.3 log_10_ CFU/0.5 mL	0.96	0.71 (evaluated for 6 *Mycoplasma* spp. and 66 species of bacteria)	[49]
multiplex qPCR Mastit 4/*M. bovis* *Staphylococcus aureus* *Streptococcus agalactiae* *Streptococcus uberis*	milk samples	-	-	-	[26]
real-time multiplex RPA/*M. bovis/uvrC* *M. haemolytica/nmaA*	deep nasopharyngeal swabs (n = 100)	40 genome copies/reaction	-	98.0 (evaluated for 10 *Mycoplasma* spp. and 35 species of bacteria)	[50]
real-time multiplex PCR PathoProof™ Mastitis Major 4.2/*M. bovis* *Staphylococcus aureus* *Streptococcus agalactiae* *Streptococcus uberis*	milk samples	-	-	-	[13]
real-time PCR VetMAX™ *M. bovis*	tissue samples, bronchoalveolar lavage fluid samples, synovial fluid, milk samples	10 genome copies/reaction	100%	100% (evaluated for 50 other bacteria species, including *M. agalactiae*, *Streptococcus uberis* and *Streptococcus dysgalactiae*	[27]
LAMP*/uvr*C, *gyr*B	milk samples from 95 dairy farms	5 × 10^1^ CFU/mL	96.8%–100%	94.7%–100% (evaluated for 2 *Mycoplasma* spp. and 4 species of bacteria)	[51]
LAMP/*opp*D	milk samples from individual quarters (n = 9); bulk tank milk samples (n = 59)	10 genome equivalents/reaction; 1 × 10^4^ cells/mL	87.5%	82.4% (evaluated for 3 *Mycoplasma* spp.)	[47]
PURE-LAMP not applicable	bulk tank milk samples (n = 12); mature milk samples (n = 73); colostrum/transitional milk samples (n = 74); mastitis milk samples (n = 122)	>10^2^ CFU/mL of milk	57.0%–97.0%	100% (evaluated for 5 *Mycoplasma* spp.)	[52]
RPA-LFD*/uvr*C, *opp*D-*opp*F	nasal swab samples (n = 288); fresh lung samples (n = 80); joint fluid samples (n = 32); bulk tank milk samples (n = 42)	20 genome copies/reaction	99.0%	95.61% (evaluated for 10 *Mycoplasma* spp. and 13 species of bacteria)	[53]
MALDI-TOF MS	culture-enriched bronchoalveolar lavage fluid samples (n = 104)	not applicable	86.6%	86.4%	[54]
